# Tackling HIV Persistence: Pharmacological versus CRISPR-Based Shock Strategies

**DOI:** 10.3390/v10040157

**Published:** 2018-03-29

**Authors:** Gilles Darcis, Atze T. Das, Ben Berkhout

**Affiliations:** 1 Laboratory of Experimental Virology, Department of Medical Microbiology, Academic Medical Center, University of Amsterdam, 1105 AZ Amsterdam, The Netherlands; gdarcis@chu.ulg.ac.be (G.D.); a.t.das@amc.uva.nl (A.T.D.); 2 Infectious Diseases Department, Liège University Hospital, 4000 Liege, Belgium

**Keywords:** HIV latency, reservoirs, cure, shock strategy, latency-reversing agents, CRISPR-Cas system, CRISPR-dCas9

## Abstract

Jan Svoboda studied aspects of viral latency, in particular with respect to disease induction by avian RNA tumor viruses, which were later renamed as part of the extended retrovirus family. The course of retroviral pathogenesis is intrinsically linked to their unique property of integrating the DNA copy of the retroviral genome into that of the host cell, thus forming the provirus. Retroviral latency has recently become of major clinical interest to allow a better understanding of why we can effectively block the human immunodeficiency virus type 1 (HIV-1) in infected individuals with antiviral drugs, yet never reach a cure. We will discuss HIV-1 latency and its direct consequence—the formation of long-lasting HIV-1 reservoirs. We next focus on one of the most explored strategies in tackling HIV-1 reservoirs—the “shock and kill” strategy—which describes the broadly explored pharmacological way of kicking the latent provirus, with subsequent killing of the virus-producing cell by the immune system. We furthermore present how the clustered regularly interspaced palindromic repeats (CRISPR) and associated protein (Cas) system can be harnessed to reach the same objective by reactivating HIV-1 gene expression from latency. We will review the benefits and drawbacks of these different cure strategies.

## 1. Introduction

Combination antiretroviral therapy (cART) allows clinicians to successfully manage the majority of human immunodeficiency virus (HIV) infected patients, to prevent the development of AIDS and to drastically reduce the risk of virus transmission. Single-tablet regimens have significantly advanced HIV management by minimizing pill burden and improving patient compliance. Unfortunately, cART is not curative. Interruption of therapy almost invariably leads to a rapid rebound of the virus to pre-cART levels in about two weeks [1,2,3,4,5,6]. Treatment therefore has to be taken for life. Clinicians have to deal with drug toxicity but also drug–drug interactions that are a growing concern since the proportion of older HIV-infected adults is increasing. Moreover, HIV infection is associated with long-term complications related to chronic immune activation and possibly premature aging. There is some evidence that effective inhibition of viral replication alone is not sufficient to restore a fully functional immune system [7]. The development of drug resistance remains another real concern. In fact, drug resistance has been documented for every currently available drug class in patients [8]. For all of these reasons, finding a cure for HIV remains a highly desirable objective.

The major hurdle to an HIV cure lies in the presence of latent HIV reservoirs. Viral reservoirs are typically defined as cell types or anatomical sites where a replication-competent form of the virus can persist, at least longer than the pool of HIV in the blood that rapidly turns over [9,10]. This definition restricts the HIV reservoirs to proviruses capable of causing viral rebound following cART interruption and which constitute the fraction of proviruses sometimes called the “true reservoir” [11]. The elimination of these proviruses should be considered in the context of a cure. Alternatively, some have recently suggested an extended definition of the viral reservoirs: all infected cells containing all forms of HIV persistence that can participate in HIV pathogenesis [12]. This broader definition includes defective proviruses that could theoretically play a role in HIV pathogenesis through the production of viral proteins and the subsequent induction of chronic immune activation [13]. In other words, proviruses that are not fully replication-competent, but that are capable of transcribing viral mRNAs and/or translating viral proteins, may constitute an additional dimension to persistence studies [14].

**Figure 1:**
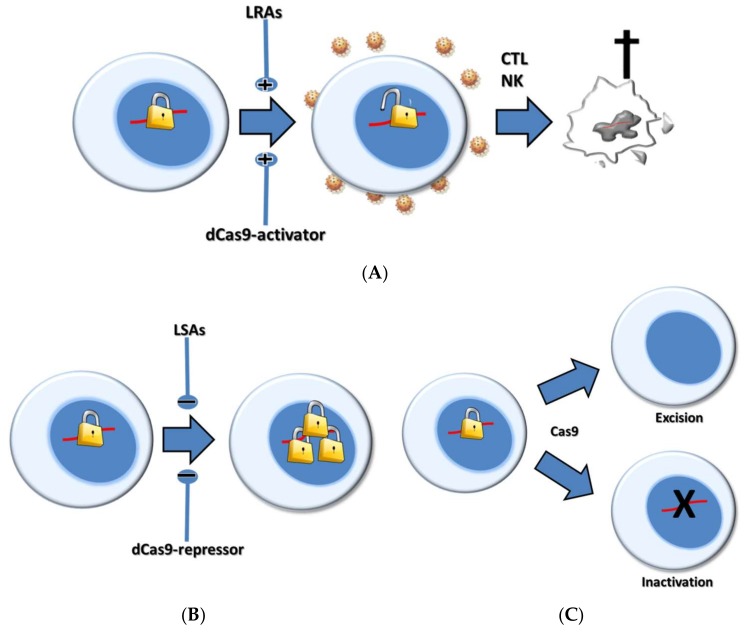
Pharmacological versus clustered regularly interspaced palindromic repeats (CRISPR)-based anti-human immunodeficiency virus (HIV) strategies. (**A**) Latency-reversing agents (LRAs) or dCas9-activators can be used to reverse HIV-1 latency (the “shock”) by activating transcription (+). This step leads to cytotoxic T lymphocytes (CTL)-mediated killing of the virus-producing cells (“the kill”); (**B**) Latency-strengthening agents (LSAs) or dCas9-repressors can be used to reinforce latency by suppressing transcription (−). This strategy should prevent viral rebound following treatment interruption; (**C**) CRISPR-Cas9 can be used to inactivate the HIV-1 proviral reservoir by excision or hypermutation.

In this review, following a brief summary on the current knowledge regarding the latent HIV reservoirs, we discuss the current therapeutic strategies aimed at targeting these reservoirs. We particularly focus on one of the most intensively explored strategies, the “kick and kill” or “shock and kill” strategy (Figure 1). We will first describe the broadly explored pharmacological interventions tested for shocking the latent provirus. We next discuss how the popular clustered regularly interspaced palindromic repeats (CRISPR) tool can be harnessed to reactivate viral gene expression from latency in a gene therapy setting, and we will review the benefits and drawbacks of each option.

## 2. The Latent HIV-1 Reservoirs

Viral persistence in CD4^+^ T cells is mostly attributed to latently-infected resting memory CD4^+^ T cells with a silent replication-competent HIV-1 genome that is not susceptible to antiretroviral drugs or immune responses. Significant progress has been made over the last few years in describing a vast functional and phenotypic diversity within these CD4^+^ T cells, in particular, the memory CD4^+^ T cell pool, and it is likely that these T cell subsets differ in their ability to serve as long-term viral reservoirs [15]. Among these subsets, central memory and transitional memory CD4^+^ T cells were identified as key populations contributing to HIV-1 persistence [16]. CD4^+^ “T memory stem cells” (TSCM) represent another memory CD4^+^ T cell subset in which long-term HIV-1 persistence is manifested, probably because of its capacity for self-renewal and prolonged survival [17,18]. Additionally, distinct lymphocyte populations within tissues, such as T follicular helper (Tfh) cells, may also serve as latent reservoirs during cART [19]. Very recently, the Tfh cell counterpart circulating in blood was shown to contribute considerably to the total pool of blood cells that contain inducible replication-competent proviruses [20]. The γδ T cells form a subset of CD3^+^ T lymphocytes that express alternative T cell receptors (TCRs) with γ and δ chains and also contribute to the pool of cells with latent, yet replication-competent, HIV-1 proviruses [21].

Overwhelming evidence supports the notion that tissues, such as the central nervous system (CNS) [22,23], lymph nodes, testes [24,25], gut [26], vaginal epithelium and lungs [27] serve as HIV sanctuaries that confound viral eradication [28]. HIV-1 infects macrophages, microglia and astrocytes in the central nervous system (CNS) [29]. The monocyte/macrophage lineage includes, among others, monocytes, macrophages, dendritic cells (DCs) and brain resident macrophages. These cells are more resistant to HIV-induced apoptosis and thus, constitute significant and stable hideouts for HIV-1 [30]. Despite the fact that only a very small percentage of monocytes harbor proviral HIV DNA, these cells are of particular importance in HIV-1 persistence due to their ability to cross the blood–tissue barrier and consequently, spread the infection into difficult to treat niches [31]. DCs also have the capacity to activate HIV-1 from latency following contact with effector T cells and thus, play a particular role in the course of HIV-1 infection [32]. 

The viral reservoirs are established very soon after the primary infection. Indeed, although the degree of latent infection can be limited by early cART initiated during acute HIV infection, treatment cannot prevent the establishment of the latent reservoir. Following intrarectal simian immunodeficiency viruses (SIV) infection of rhesus macaques, the viral reservoir is seeded as early as three days post-infection, thus well before viremia is detectable in the mucosal and lymphoid tissues that form the first sites of viral replication [33]. Henrich and colleagues recently reported two cases of extremely early initiation of ART following the “eclipse phase” in Fiebig stage I [34]. Despite treatment being started only a few days after infection, a small HIV reservoir size was measured in the study participants [34]. Early treatment undoubtedly has a favorable impact on the HIV-1 reservoir size and distribution, as highlighted by the Visconti study [35], but unfortunately, it cannot preclude its establishment [36].

The heterogeneous HIV-1 reservoirs are thus established very early during the course of HIV infection and remain remarkably stable over a prolonged period, despite suppressive cART. It has been estimated that, due to the very long half-life of the latently effected cells, 70 years of constant therapy would be required for complete removal of the reservoirs. Additionally, homeostatic proliferation and clonal expansion may play a critical role in maintaining or even increasing the reservoir size [37]. These processes obviously further complicate the composition of the HIV reservoir and its dynamics.

Several cure strategies have been developed in attempts to eradicate the HIV-1 reservoirs, together with sensitive assays aimed at measuring these reservoirs that are needed to measure the impact of the cure strategies [38,39,40,41]. One of the most popular cure strategies is based on a reversal of HIV latency in patients on cART: “the shock”. Cells harboring such induced proviruses will subsequently be eliminated by the viral cytopathic effects or host immune effector mechanisms—“the kill”—while infection of bystander cells would be impeded by ongoing cART. Huge progress has been made in understanding the mechanism of HIV-1 latency and its determinants. HIV-1 latency can be established (and sustained) through different molecular mechanisms that operate at the transcriptional level or at the post-transcriptional stage of virus production, including HIV mRNA export from the nucleus, splicing and translation into viral proteins. Understanding of the underlying molecular mechanisms has also allowed the development of small molecule drugs that are capable of inducing HIV expression, or at least viral transcription. These molecules, some of which were already used in clinical practice, especially in the cancer field, are called latency-reversing agents (LRAs) [42]. Alternatively, in recent years, several transcription-activating CRISPR-based systems have been developed with the objective of potently reactivating HIV-1 gene expression in a sequence-specific manner (Figure 1). Interestingly, small molecules (named here as latency-strengthening agents—LSAs) as well as CRISPR-based systems have also been developed for the opposite goal—the reinforcement of viral latency—a strategy that would impede HIV-1 production from the viral reservoirs and would therefore reduce the associated immune chronic activation (Figure 1). This specific topic has been reviewed elsewhere [9]. In the following sections, we describe, in more detail, the pharmacologic and CRISPR-based shock strategies and their respective advantages and disadvantages. 

## 3. Pharmacologic Shock Strategies

### 3.1. Molecular Mechanisms of HIV Latency

HIV-1 latency can be established (and maintained) by different mechanisms that operate at the transcriptional level. Transcriptional regulation of HIV-1 gene expression and latency results from a complex and dynamic interplay of multiple factors that act at the initiation, but mostly the elongation phase of transcription [43,44]. The underlying molecular mechanisms are only partially understood and remain the focus of intense research, including topics like epigenetic modification of the HIV-1 long-terminal repeat (LTR) promoter, the absence of transcription factors or the presence of repressive factors.

It has been well recognized that epigenetic modification of histone tails at the HIV-1 LTR promoter is important for the establishment of latent reservoirs [45]. Reversible epigenetic marks affect gene expression by modifying chromatin condensation and consequently, the accessibility of the HIV-1 promoter to transcription factors. These epigenetic modifications include histone acetylation/methylation and DNA methylation, which are catalyzed by enzymes such as histone acetyltransferases (HAT), histone deacetylases (HDAC), DNA methyltransferases (DNMT) and histone methyltransferases (HMT). Histone crotonylation has been implicated in the regulation of host gene expression, and a recent report suggested that this novel epigenetic mark participates in the regulation of HIV transcription [46].

Acetylation of lysine residues in histone tails favors chromatin opening and thereby, increases the accessibility of the HIV promoter for binding of transcription factors. In addition to this direct effect, histone acetylation also triggers the binding of bromodomain-containing proteins that regulate gene expression [47]. In contrast, deacetylation by HDAC creates a tightly packed DNA form that does not support high-level transcription [47,48].

Histone methylation can be associated with transcriptional repression or activation. Trimethylation of the ninth lysine of the N-terminal tail of histone 3 (H3K9Me3) and H3K27 trimethylation (H3K27me3) repress gene expression and have been associated with HIV-1 latency in different post-integration latency models [49,50]. The HMT enhancer of Zeste homolog 2 (EZH2), which is required for H3K27me3 modification, is present at high levels in the LTR promoter of silenced HIV-1 proviruses [50]. SET and MYND domain-containing protein 2 (SMYD2), a member of the SMYD family of methyltransferases, is another HIV-1 transcriptional repressor that acts through methylation of H3K36 and H3K4 [51,52,53].

DNA methylation leads to a repressive chromatin structure and could participate in the maintenance of HIV latency [54]. Remarkably, DNA methylation of the HIV-1 promoter in patient cells increases over time during cART treatment, highlighting that HIV-1 persistence is a dynamic process [54]. 

Although a lot remains to be done to unravel the mechanisms leading to the establishment and maintenance of these epigenetic factors, several reports recently showed that an HIV-1 antisense transcript expressed from the 3′LTR promotes proviral latency through the recruitment of DNMT3a, EZH2 and HDAC1 to the HIV-1 5′LTR promoter region [55,56,57]. These reports are of particular importance since they involve the possibility that viral mechanisms of latency could be specifically targeted to reverse or reinforce HIV latency.

Other key molecular mechanisms involved in HIV-1 latency are sequestration of transcription initiation/elongation factors and blockage of the assembly of the active elongation factor, P-TEFb. The lack of active forms of relevant cellular transcription factors is a major factor in the repression of HIV transcription at the initiation and elongation levels in resting CD4^+^ T cells [58]. For instance, NF-κB is sequestered in an inactive form in the cytoplasm of resting cells through interaction with the inhibitor of NF-κB (IκB). Cellular activation leads to IκB phosphorylation and subsequent proteosomal degradation. NF-κB then migrates into the nucleus to activate NF-κB-responsive genes, including the HIV-1 LTR promoter that contains two NF-κB binding sites. Additionally, resting CD4^+^ T cells are characterized by a low level of P-TEFb, a crucial elongation factor composed of cyclin T1 and CDK9 [59,60,61]. P-TEFb is sequestered in an inactive complex but can be released as an active P-TEFb complex following activation of the cells by various stimuli [62]. Recently, it has been shown that knockdown or inhibition of the mammalian target of the rapamycin (mTOR) complex suppresses HIV-1 transcription via blocking CDK9 phosphorylation [63]. This finding further supports the important function of the P-TEFb complex in HIV-1 transcription.

### 3.2. Latency Reversing Agents

Understanding the nature of HIV-1 persistence has allowed the identification and testing of a wide variety of latency reversing agents (LRAs) that act on different mechanisms of latency. Interestingly, some LRAs are already in clinical use in the cancer field or in the treatment of medical conditions, like alcohol dependency and epilepsy [64,65,66]. This allowed us to quickly perform clinical studies following encouraging in vitro and ex vivo results for some of these LRA candidates (Table 1).

Stimulation of latently-infected cells with HDAC inhibitors (HDACIs), the most studied LRAs, has been described for both in vitro and ex vivo cultures of primary cells from cART-treated HIV-1^+^ individuals [67,68,69,70,71]. Some of these HDACIs were subsequently tested in vivo in pilot clinical trials and were able to reverse HIV-1 latency to a variable degree [72,73,74,75,76]. 

Disulfiram, an FDA-approved drug used to treat alcohol dependence, has been identified as a potential LRA that acts through induction of the AKT pathway [77]. Two clinical trials have tested this drug as an LRA, showing only a very modest ability to overcome HIV-1 latency in vivo [64,78]. 

The NF-κB pathway appears to be one of the most relevant pathways concerning HIV-1 reactivation. Protein kinase C agonists (PKCA) activate PKC isoforms which then phosphorylate IκB and consequently, activate NF-κB. Several PKC agonists have been considered for purging the reservoirs of latent HIV-1. The interest in this LRA class has been triggered by their ability to reactivate HIV-1 across different experimental latency models [79], including three independent ex vivo assays [80]. Bryostatin-1 has been tested in a pilot clinical trial in 12 aviremic HIV-1-infected patients on cART. However, the drug did not have any effect on PKC activity or transcription of latent HIV, probably due to the low plasma concentration [81]. 

Toll-like receptor (TLR) agonists are also under investigation as potential LRAs. TLR agonists are key players of the cellular innate immune system that recognize pathogen-associated molecular patterns. TLR agonists are also functionally present in CD4⁺ T cells. The TLR-5 agonist, flagellin, the TLR-1/2 agonist, Pam3CSK4, and the TLR7-selective agonist, GS-9620, have been successfully tested as LRAs in vitro and ex vivo [82,83,84]. The TLR9 agonist, MGN1703, was subsequently tested in a clinical trial, where it both enhanced innate immunity and induced plasma HIV-1 RNA blips up to >1500 copies/mL in a subset of participants [85]. However, this did not result in a reduction of the HIV-1 reservoir size, as was assessed through the measurement of total HIV-1 DNA and integrated HIV-1 DNA, but also by the quantitative viral outgrowth assay [85]. 

Clinical trials of individual LRAs have yielded variable, but sometimes encouraging results concerning their ability to induce HIV-1 transcription [86]. However, none of these studies have caused significant and persistent reduction of the HIV-1 reservoir size. The causes of this ineffectiveness, some of which are listed in Table 1, have to be understood in order to rationally improve the “shock” strategy so that it reaches clinical success. 

The lack of selectivity is undoubtedly a major obstacle towards a safe and efficient HIV-1 reversal from latency. As explained above, most of the known molecular mechanisms of HIV-1 transcriptional repression are general cellular mechanisms that are not specific to HIV-1 (Table 1). The pharmacologic drugs used to reactivate latent HIV-1 so far nonspecifically target cellular mechanisms, which could lead to adverse effects. For instance, the promising bryostatin-1 drug has been associated in vivo with dose-dependent myalgia [87]. The lack of specificity of LRAs may also trigger unwanted reactivation of other latent viral pathogens (e.g., cytomegalovirus and Epstein–Barr virus) (Table 1).

Another emerging hurdle in pharmacologic latency reversal is the heterogeneous and dynamic nature of the cellular mechanisms involved, which may vary considerably from cell to cell [88]. Yucha and colleagues developed a single-cell assay to identify and quantify transcriptionally active cells and their responses to various ex vivo stimuli, including LRAs. They observed much variation in the single-cell response to global TCR stimulation and HDACi exposure between different individuals as well as within cells from a single individual [89]. It is also important to stress that the proviral responsiveness to LRAs can also vary by integration site. Chen et al. recently reported that phytohemagglutinin and the HDACI vorinostat reactivate specific proviruses which differ from one another by their genomic integration site, suggesting that the chromosomal context is a critical determinant of the viral response to reactivation therapies [90]. To create a more general and robust therapeutic effect, one could consider the combined use of multiple LRAs. Several groups have explored LRA combinations in vitro and ex vivo and reported encouraging synergistic effects (e.g., HDACI + PKCA combined treatment [91], JQ1 + PKCA [62,92], DNMTI + HDACI [66] and more recently, TLRA + PKCA [93]; see [94] for a complete overview). Clinical trials aimed at targeting the HIV-1 reservoir with LRA combinations are definitely needed. 

The inability of the pharmacological shock strategy to minimize the HIV-1 reservoirs may also be due to the fact that “the shock” is not followed by “the kill” [95]. A recent report suggested that latently-infected cells harboring an infectious provirus might even display a higher resistance to CD8^+^ T cell–mediated killing than cells with a defective HIV provirus [96]. The reasons for this difference are still poorly understood. Deng et al. showed that unless cART is started early, the vast majority of latent proviruses will encode cytotoxic T lymphocytes (CTL) escape mutations that render these cells insensitive to CTL-directed killing. Several reports have pointed out that LRAs could also have effects on the innate and adaptive immune responses. Jones et al. reported that the HDACIs—vorinostat, romidepsin and panobinostat—impair the CTL-mediated elimination of HIV-infected cells [97]. Walker-Sperling et al. confirmed the inhibitory effects of romidepsin on the suppressive capacity of HIV-specific CD8^+^ T cells and extended this negative impact to bryostatin-1 [98]. These inhibitory effects were not apparent for prostratin and ingenol-B [98,99]. Interestingly, Garrido et al. also studied the impact of several HDACIs and PKCAs on the function of natural killer (NK) cells and reported that some LRAs alter their function [100]. To make the pharmacologic shock strategy effective, it is important to address these issues. The combination of LRAs to reach a more robust latency reversal could be one approach. The addition of some form of immune boosting to optimize “the kill” is currently also under intense investigation [101]. 

## 4. CRISPR-Based Shock Strategies

Potent therapeutic regimens based on the RNA interference (RNAi) and CRISPR-Cas mechanisms have been developed over the last decade [102,103,104,105,106]. These mechanisms attack different forms of the viral genome, RNA and DNA, respectively, but both mechanisms act in a strictly sequence-specific manner, which make them ideal for therapeutic action [107]. Some of these therapeutic tools have also been used to impair CCR5 expression and to block HIV entry [108,109]. The CRISPR system, extensively used as an effective gene-editing tool, has also been harnessed to reactivate HIV-1 gene expression from latency (Figure 1). In this section, we review the literature related to this specific application of the CRISPR system and explain why this system could address some of the hurdles in achieving an effective pharmacologic shock strategy. 

The CRISPR system, comprised of a short guide RNA (gRNA) and the Cas9 endonuclease, has been used in anti-HIV strategies, mostly as a genome editing tool that can cleave double-stranded HIV DNA in a sequence-specific manner (reviewed in [110]). This cleavage is immediately followed by DNA repair by the non-homologous end joining (NHEJ) mechanism, which introduces mutations (mostly deletions and insertions; indels) into the target sequence. HIV can escape when a single gRNA is used, but some combinations of two gRNAs have been shown to prevent viral escape [111,112]. Such dual gRNA treatment can trigger complete HIV-1 inactivation, either by mutation at both target sites or by deleting the viral fragment located between the two target sites [111,113,114,115,116] (Figure 1). The gRNA viral target sequences should always be carefully designed in order to prevent damage in the cellular DNA, which could have severe adverse effects. The CRISPR system has proven its ability to attack the proviral DNA reservoir directly, although delivery to all relevant cells remains a major challenge.

The exquisitely sequence-specific CRISPR system can also be modified for a new task of HIV purging. Mutation of the Cas9 catalytic domain generates a “deactivated” or “death” Cas9 (dCas9). This dCas9 can be used for several gRNA-mediated DNA-targeting purposes, including activation of gene expression by fusion of the dCas9 protein to a strong transcription activation domain (AD). Targeting of these dCas9-AD proteins to the HIV-1 LTR promoter will attract transcription activating and chromatin modifying factors that can activate viral transcription. This strategy seems a priori more safe than regular Cas9 cleavage action due to the absence of DNA damage to the host cell, but the jury is out in regard to the potential of off-target gene activation events. 

Several dCas9-AD systems that use ADs from viral or cellular transcription factors have been developed. The dCas-VP64 protein, which contains four copies of the herpes simplex virus (HSV) VP16-derived minimal AD, has been tested for activation of HIV-1 LTR-driven gene expression in different cell lines with episomal and integrated LTR reporter constructs and in latently infected cells [117,118,119,120,121]. The position of the gRNA target in the LTR promoter influenced the efficiency of activation, and most studies have identified the NF-κB binding sites as the most promising target regions [121]. Activation of gene expression could be enhanced by combining multiple gRNAs with non-overlapping targets, thus recruiting multiple copies of dCas9-VP64—and thereby more transcription factors—to the LTR promoter [122]. 

Several alternative dCas9-AD systems that were designed to more potently activate gene expression have been tested on the HIV-1 LTR promoter. The dCas-VPR fusion protein contains not only four VP16 Ads, but also an NF-κB trans-activating subunit p65 and the Epstein–Barr virus R transactivator domain (Rta) to attract additional transcription factors [123]. The “synergistic activation mediator” (SAM) system uses a modified gRNA with binding sites for MS2 bacteriophage coat protein [124]. This gRNA will not only attract dCas9-AD fusion proteins, but also MS2-AD fusion proteins, like the MS2-p65-HSF1 protein, consisting of MS2, p65 and the human heat shock factor 1 AD. In the dCas9-SunTag system, dCas9 is fused to multiple copies of a GCN4 peptide and co-expressed with a GCN4-binding single-chain variable-fragment (scFv) antibody fused to four VP16 ADs [125]. The dCas-SunTag protein will thus form a scaffold that attracts a large number of ADs. Most studies in which the efficiency of the original dCas9-VP64 system was compared to that of the dCas9-VPR, dCas9-SAM or dCas9-Suntag systems demonstrated an improved activity of the latter systems [117,120,121]. For example, Ji et al. showed that dCas9-SunTag could reactivate latent HIV-1 significantly better than dCas9-VP64 in different latently-infected cell lines [126], and Limsirichai et al. showed that dCas9-SAM activates the HIV-1 LTR more potently than dCas9-VP64 in transient reporter assays [120]. However, different levels of dCas9-SAM mediated activation of HIV-1 gene expression were observed when testing several J-Lat cell lines that differed in the site of HIV-1 genome integration. This latter result indicates that epigenetic or genetic factors can influence the capacity of dCas9-AD systems to reactivate HIV-1 transcription [120]. In the same J-Lat latency model, epigenome-modifying technologies have the potential to directly alter HIV-1 gene expression. The dCas9-p300 system, in which dCas9 is fused to the histone acetyltransferase core domain of the human E1A-associated protein, p300 [119], will catalyze histone acetylation at the gRNA target site, which can activate transcription when promoter or enhancer sequences are targeted. Indeed, dCas-p300 targeting of the viral LTR can reactivate HIV-1 transcription in J-Lat 10.6 cells [120]. Interestingly, using J89 latently infected cells carrying a fully replication-competent HIV-1 provirus, Bialek et al. demonstrated that robust dCas-SAM-mediated activation of HIV-1 transcription can induce the production of infectious viral particles [118]. The dCas-SAM system thus seems to have the ability to induce HIV-1 reactivation in vitro in a way that causes transcriptional and any post-transcriptional blocks to be overcome. Such a robust and persistent reactivation of latent HIV-1 proviruses could induce cell death (“the kill”) in various post-integration latency models of monocytic and lymphocytic origin [117]. 

## 5. Pharmacological Versus CRISPR-Based Shock Strategies

The innovative CRISPR-based reactivation strategy holds several advantages over the LRA-based approach, but also some pertinent disadvantages (Table 1). The high sequence-specificity of Cas9-action certainly creates a major specificity benefit. The gRNAs should be carefully designed to avoid off-target effects on cellular genes. Most previous studies have shown the CRISPR-based strategy to have a higher reactivation potency, compared to single LRAs [117,120,121,126]. In addition, CRISPR was able to induce infectious virus particle production [117], whereas most LRAs failed to do so [80]. Last but not least, CRISPR-based reactivation may be linked to death of the treated cell [117]. This effect may relate to the potency of HIV-1 production triggered by CRISPR. The CRISPR-based strategy would also avoid the LRA-mediated effects on innate and adaptive immunity. 

However, in contrast to the pharmacological-based reactivation strategy that has reached patients over the past few years, the CRISPR approach is still relatively far from clinical testing. Most CRISPR studies focus on diverse in vitro cell culture models, including various latency models, and only few studies have yet been performed in vivo in murine models [114,127]. Successful translation to clinical HIV trials necessitates the effective and safe delivery of dCas9-AD and gRNA to most, if not all, infected cells. Although several methods are available for transient delivery of these components, viral vector delivery methods, like the adeno-associated virus (AAV) and especially, the HIV-based lentiviral vector (LV) systems, may provide more sustained HIV-1 activation. A key restriction in the viral delivery of the CRISPR-based system is the transgene size since viral vectors have limited ability to store genetic information, whereas current dCas9-AD genes are based on the 4.2 kb Cas9 of *Streptococcous pyogenes* Cas9. Indeed, neither dCas9-SAM nor dCas9-P300 could be packaged efficiently into a single AAV vector [120]. Split-dCas9 systems, in which different domains of dCas9 are encoded by separate vectors, can be considered but will likely be less effective, e.g., because all vectors have to reach the same target cells. The use of smaller dCas9 genes based on Cas9 variants from other bacterial species or truncated dCas9 proteins lacking non-essential domains can also be explored [128]. 

Potential off-target effects remain a serious concern for the clinical application of the CRISPR-based dCas9-AD system, although to a lesser extent than for the DNA-cleaving Cas9 variant. The gRNAs should be carefully selected with bioinformatic tools and subsequently, extensively tested to check for high on-target and no off-target activity [129]. Finally, the bacterial origin of the CRISPR-based systems might incite detrimental host reactions, including immune reactions that may neutralize the therapeutic effect over time [130]. Moreover, delivery by means of adenovirus vectors can also cause severe inflammation and immunological reactions [131]. Immunologically inert delivery vectors are hence particularly attractive. Clinical implementation of the CRISPR-based system therefore requires comprehensive evaluation of its in vivo activity, including adverse effects. Both pharmacologic and CRISPR-based approaches may thus cause unintended side effects. The pharmacologic approach has the advantage that can be promptly interrupted, thus limiting detrimental outcomes. In this regard, it has to be noted that CRISPR-based system activity can be regulated, for instance, when Cas9 or gRNA expression is controlled by a doxycycline-inducible promoter [132,133,134,135]. Cas9 activity can also be regulated at the post-transcriptional level [136]. However, this approach would most likely not impede any Cas9-induced host immunological reaction. 

## 6. Conclusions

The “shock and kill” strategy for purging latent viral reservoirs is one of the most explored approaches in reaching a cure for HIV. This approach is based on HIV reactivation in latently-infected cells, followed by the death of these virus-producing cells. Understanding some of the molecular mechanisms involved in inducing and controlling HIV-1 latency has led to the identification of LRAs, small molecule drugs that have already been developed for other medical conditions. Several LRAs have already been tested in terms of their potential to purge latent HIV in vivo in clinical trials, but so far failed to reduce the viral reservoirs. The heterogeneity of these reservoirs, the inability of the immune system to clear the LRA-activated reservoirs, and the lack of specificity of these LRA drugs are some of the many emerging barriers in developing an efficient pharmacologic shock strategy. The use of compounds combining shock and immunomodulatory capacities, such as TLR agonists, can address some of these hurdles. Combining LRAs with immune boosting to optimize the kill is also a strategy that deserves further investigation. The CRISPR-based shock strategy forms an attractive, alternative therapeutic approach. CRISPR allows a very specific and potent HIV reactivation, followed by the death of virus-producing cells. Although promising, many challenges remain, including efficient in vivo delivery of the CRISPR system and the potential long-term toxicity. We realize that there is still a long and bumpy road ahead of us, riddled with major hurdles, before this strategy can be evaluated in clinical trials. Finally, the combination of pharmacologic and CRISPR-based approaches has provided encouraging results regarding HIV reactivation [120]. However, combining both strategies also implies the need to address many issues related to both approaches and consequently, does not appear to be the shortest route in finding a cure for HIV.

## Figures and Tables

**Table 1:** Strengths and weaknesses of pharmacological and CRISPR-based shock strategies.

Shock Strategies to Cure HIV-1	
Latency-Reversing Agents	CRISPR-dCas9
Not specific	Sequence-specific
Toxicity at a high dose	Possible off-target effects not yet known
Weak as individual drugs	Potent (in vitro)
Already tested ex vivo and in vivo	Only tested in vitro thus far
Possible effects on other latent viruses (e.g., EBV, CMV)	Effect on other viruses unlikely
Diffusion via blood into the cells	Transduction of the multiple components required
Impact on immune system?	Host immune response due to bacterial origin

EBV: Epstein–Barr virus, CMV Cytomegalovirus.
